# Tuning a secondary dose verification software for a CT‐guided online adaptive delivery system

**DOI:** 10.1002/acm2.14563

**Published:** 2024-11-29

**Authors:** Xiaodong Zhao, Markus Baur, Phillip D. H. Wall, Eric Laugeman

**Affiliations:** ^1^ Department of Radiation Oncology Washington University in St. Louis St. Louis Missouri USA

**Keywords:** Ethos, Mobius3D‐Adapt, online QA

## Abstract

**Background:**

Quality assurance (QA) remains unstandardized for CT‐guided online adaptive radiotherapy (CTgART) platforms (Ethos, Varian Medical Systems, Inc., Palo Alto, CA), as they become more clinically prevalent. A secondary dose calculation software (Mobius3D, Varian Medical Systems, Inc., Palo Alto, CA) is provided for this closed CTgART system. However, the clinical impact of tuning dosimetric leaf gap (DLG) correction values for specific delivery techniques for CTgART secondary dose calculations remains uninvestigated.

**Purpose:**

Tuning the DLG correction value for different delivery techniques of the independent secondary dose verification software for adaptive online QA.

**Methods:**

A total of 31 volumetric arc therapy (VMAT) and 13 fixed‐gantry intensity modulated radiation therapy (IMRT) plans were selected from representative anatomical sites treated in our clinic. All plans were evaluated on a patient CT dataset and a QA dataset of a solid water phantom with an embedded ion chamber placed at the center in both primary treatment planning systems (TPS) and secondary dose verification software. Primary TPS plan doses were compared with secondary calculation doses on patient CT by calculating 3D gamma passing criteria under different DLG correction values ranging from –2 to 2 mm to determine the optimal DLG correction range. Ion chamber verification measurements were then compared to secondary calculation dose to determine the optimal DLG correction value by minimizing the difference for IMRT and VMAT plans, separately.

**Results:**

The optimal DLG correction values for VMAT and IMRT techniques were –0.3 and 0.4 mm respectively for the selected clinical cohort of patients. The mean gamma passing rate between primary and secondary doses for VMAT and IMRT were 99.0% ± 1.0% and 99.9% ± 0.1% with 3%/2 mm and excluding 10% low dose criteria. The mean ion chamber agreement for VMAT and IMRT were 0.0% ± 2.1% and 0.2% ± 1.4%.

**Conclusion:**

DLG correction value should be tuned for each delivery technique (VMAT and IMRT) separately to maximize the robustness of CTgART online QA programs.

## 1 INTRODUCTION

Online adaptive radiotherapy (ART) is becoming increasingly prevalent due to its ability to adapt treatment plans to the patient anatomy of the day. The Ethos platform (Varian Medical Systems, Inc., Palo Alto, CA) utilizes high‐quality kV‐cone beam computed tomography (CBCT) imaging with the Halcyon ring gantry linear accelerator to enable CT‐guided online adaptive radiotherapy (CTgART).^1^, ^2^, ^3^, ^4^ Since the patient remains in the treatment position during online adaptive planning sessions, pre‐treatment patient‐specific quality assurance (PSQA) must be performed via an independent secondary dosimetry system in lieu of traditional measurement‐based methods. Physicists typically review and approve the plan in both the primary and secondary dosimetry systems for online treatments.^5^, ^6^

To our knowledge, the only commercially available secondary dosimetry system provided for this closed system is Mobius3D‐Adapt (Varian Medical Systems, Inc., Palo Alto, CA). Mobius3D‐Adapt is a module within Mobius3D (Varian Medical Systems, Inc., Palo Alto, CA), which has its own Digital Imaging and Communications in Medicine (DICOM) daemon that communicates with the treatment delivery machine during online sessions for secondary dose calculations. In general, Mobius3D recalculates the dose on the patient CT dataset using an independent beam model with a collapsed‐cone convolution/superposition (CCC) dose calculation algorithm with the DICOM‐RT Plan file generated from the clinical treatment planning system (TPS).^7^

The commissioning of Mobius3D for Ethos is similar to other primary or secondary dose calculation algorithms. Once open‐field dosimetry and absolute dose calibrations are verified, simple (e.g., 3D conformal) and complex (e.g., intensity modulated) treatment plans are subsequently tested and verified.^8^ However, most beam model parameters for Ethos in Mobius3D are not user‐adjustable except the multileaf collimator dosimetric leaf gap (DLG) correction value from the Mobius3D default.^9^ The DLG correction value works as a fine scaling factor. “Positive DLGs result in higher doses, while negative DLGs result in less doses^9^”. There are two delivery techniques available in versions 4.0.2 and above where the DLG correction can be assigned individually: default (fixed‐gantry intensity modulated radiation therapy [IMRT]) and volumetric arc therapy (VMAT). This ostensibly allows the user to adjust the DLG correction value in Mobius3D to better fit measurements separately for IMRT and VMAT.

Validation of Mobius3D on different treatment platforms with different machine configurations, treatment modalities, and calculation algorithms has been studied previously.^10^, ^11^, ^12^, ^13^, ^14^ To our knowledge, experience in tuning different DLG correction value per specific delivery techniques for Halcyon/Ethos has yet to be reported. Therefore, the purpose of this study is to comprehensively analyze the impact of the Mobius3D DLG correction value on the performance of its dose calculation for different treatment modalities on a ring‐gantry Linac. This study also presents best‐fit DLG correction results for IMRT and VMAT plans treated on Ethos with the double‐stacked multileaf collimator (MLC) model in a diverse patient cohort at our institution.

## 2 METHODS

The QA outcomes of 44 patients previously treated at our institution—with both adaptive radiotherapy and non‐adaptive therapy using the Ethos treatment delivery system—were analyzed. Of these 44 cases, 31 were VMAT deliveries and 13 were fixed‐gantry IMRT plans. Patients from the past 3 years were specifically selected to represent the relevant range of treatment sites and modalities treated and utilized in our clinic, as shown in Table 1.

**TABLE 1:** Summary of treatment sites and modalities.

Anatomical treatment site	Dose/fractionation	Delivery technique	Number of plans
Head & neck	conventional	IMRT	4
Bladder	conventional	IMRT	5
Prostate	SBRT^a^	IMRT	1
Lymph nodes	SBRT^a^	IMRT	3
Brain	conventional	VMAT	2
Head & neck	conventional	VMAT	4
Accelerated partial breast irradiation	SBRT^a^	VMAT	5
Stomach	SBRT^a^	VMAT	1
Pancreas	SBRT^a^	VMAT	1
Liver	SBRT^a^	VMAT	3
Kidney	SBRT^a^	VMAT	1
Abdominal/Pelvic mass	SBRT^a^	VMAT	4
Lymph nodes	SBRT^a^	VMAT	8
Bladder	conventional	VMAT	1
Pelvis	conventional	VMAT	1

Abbreviations: IMRT, intensity modulated radiation therapy; VMAT, volumetric arc therapy.

^a^ SBRT here indicates the dose per fraction was over 500 cGy.

3D gamma analysis between primary TPS calculated dose to Mobius3D calculated dose on patient CT was performed in Mobius3D version 4.0.2. The primary TPS used in the study was Eclipse version 15.6 with Acuros XB (AXB) algorithm version 15.6.06 (Varian Medical Systems, Inc., Palo Alto, CA). Ethos AXB has shown to be very similar to Eclipse AXB from previous publication^15^ and Eclipse was used due to the ease of dose‐comparison needed in this study, particularly in generating ion chamber‐based QA plans. The DLG corrections for both IMRT and VMAT were initially selected at [–2, 2] mm in 0.5 mm increments. The range of optimal DLG was narrowed down to a 0.5 mm range [–0.5, –0.1] for VMAT and a 0.6 mm range [0.3, 0.8] for IMRT based on the 3D gamma passing rate for subsequent ion chamber comparison. The dose calculation resolution in primary TPS was 2.5 mm x 2.5 mm x 2 mm and in the secondary verification software was 2.5 mm x 2.5 mm x 2.5 mm. Passing thresholds for 3D gamma was defined at a 95% passing rate using 3%/2 mm dose‐difference/distance‐to‐agreement criteria, while excluding dose points below 10% of the global maximum in accordance to department clinical protocols and recommendations from TG‐218.^16^ Warning and failing thresholds for 3D gamma analyses were 90%–95% and less than 90%, respectively.

**FIGURE 1:**
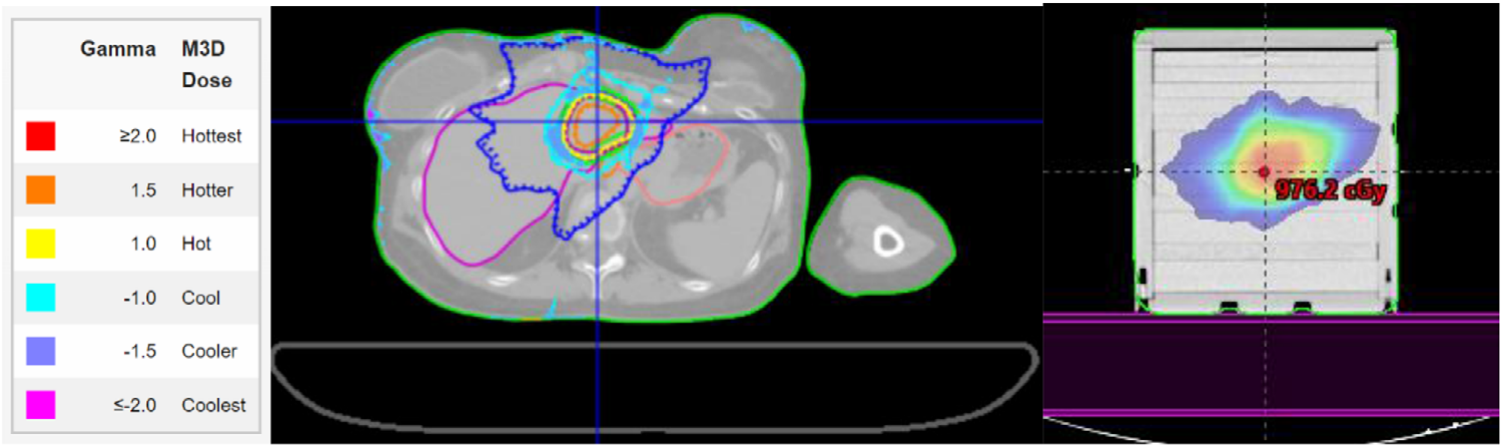
Schematic diagram of Mobius3D calculated dose on patient CT (Left) and ion chamber measurement setup in solid water QA phantom (Right). In Mobius3D, solid and dashed lines are isodose lines from primary TPS and Mobius3D, respectively. Structures including targets and ROIs are overlaid on the 3D image set as well. Failing gamma values are shown as shaded areas of different color based on the legend on the left to visualize regions that are flagged by Mobius3D. QA, quality assurance; ROIs, region of interests; TPS, treatment planning systems.

Ion chamber evaluation was done subsequent to these gamma analyses. The PSQA plans were generated in Eclipse with the AXB dose calculation algorithm. A 0.125 cm^3^ ion chamber (PTW N31010 PTW Dosimetry, Freiburg, Germany) embedded in a 17cm×17cm×17cm solid water phantom was utilized for measurement. The chamber was cross‐calibrated with an Accredited Dosimetry Calibration Laboratories‐calibrated chamber in the solid water prior. The isocenter was shifted in the phantom so that the measurement point was selected to be within high dose region (> 80% of prescription dose) without sharp dose gradients (< 5% of dose deviation). The mean expected dose from the ion chamber volume contour was compared to the measured dose. The measurement was corrected for daily output variation with an open field. kV planar image and CBCT were used to align the phantom before measurement. Then couch shifts were applied based on the PSQA plan created in TPS. The PSQA plans were then exported to the Mobius3D for ion chamber evaluation as shown in Figure 1.

The same PSQA plans were recalculated with varying DLG correction values in Mobius3D and the Mobius3D‐calculated ion chamber dose was recorded. A 2.5% agreement between the ion chamber measurement and the given calculated dose was defined as the passing threshold per institutional guidelines. Warning and failing criteria were 2.5%–3.0% and greater than 3.0% for ion chamber measurement, respectively. Subsequently, the agreement between the measured ion chamber dose and the calculated Mobius3D dose was evaluated to find the best‐fit DLG correction values for the given delivery technique (VMAT or IMRT). Ion chamber measurement results were compared to the primary TPS calculated dose on the solid water phantom as well for reference.

To test the statistical significance of the optimal DLG correction, linear regression models are fitted for each delivery technique (VMAT or IMRT). A linear regression model of DLG correction as a function of the ion chamber percent difference between measurement and Mobius3D, and gamma passing rate between primary TPS and Mobius3D is considered. The predictors used include gamma median, gamma min, and ion chamber mean. *p*‐values for the *F*‐test on the models are presented.

## 3 RESULTS

**FIGURE 2:**
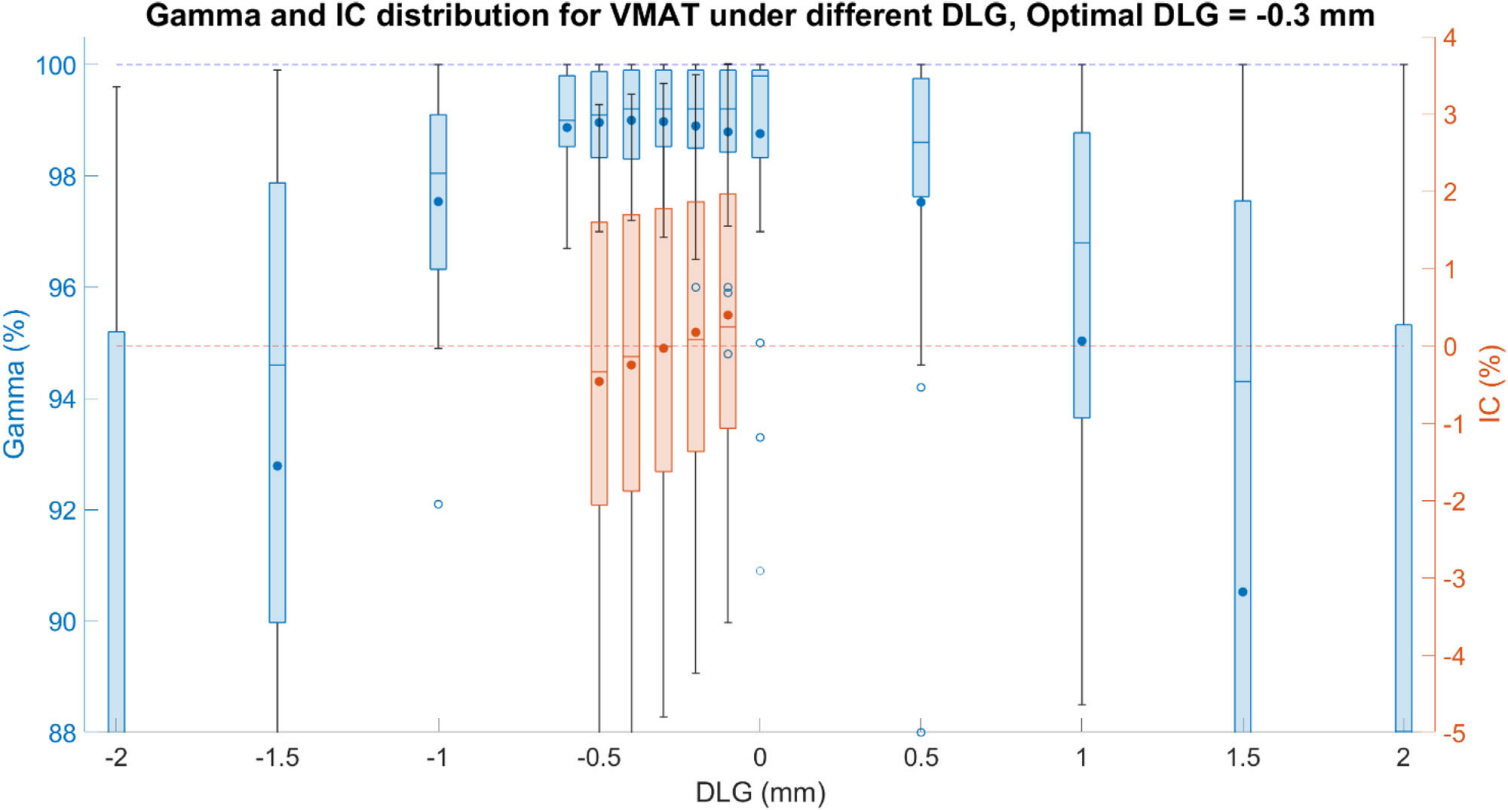
The 3D gamma passing rate between primary TPS and Mobius3D and IC agreement distribution between measurement and Mobius3D under different DLG correction settings for VMAT plans. The 3D gamma was used as an initial DLG correction range estimate so it is shown with a wider DLG correction range. The box plots show the median, the lower and upper quartiles, and any outliers, with solid dots in each box representing the mean value. The blue plots represent the 3D gamma passing rate and the red plots are ion chamber agreement distribution. The dotted blue or red lines indicate designed goals, which are 100% and 0% agreement. DLG, dosimetric leaf gap; IC, ion chamber; TPS, treatment planning systems; VMAT, volumetric arc therapy.

Mobius3D gamma passing rate and the ion chamber measurement results for VMAT plans with varying DLG corrections are shown in Figure 2. The best‐fit DLG correction for VMAT was –0.3 mm, where the mean and standard deviation of ion chamber results were 0.0% ±  2.1% and the mean and standard deviation of 3D gamma was 99.0% ±  1.0% with a range of [96.9%, 100.0%]. For DLG = –0.3  mm, all plans had gamma passing rates of over 95%, and all but four plans had ion chamber agreements within 3%. The four ion chamber outliers were within 5% of measurement to Mobius3D calculated dose, and these four ion chamber measurements to primary TPS dose were all within 1.5%. The mean and standard deviation of ion chamber measurements to primary TPS was –0.6% ±  1.0% and the median was –0.5%.

**FIGURE 3:**
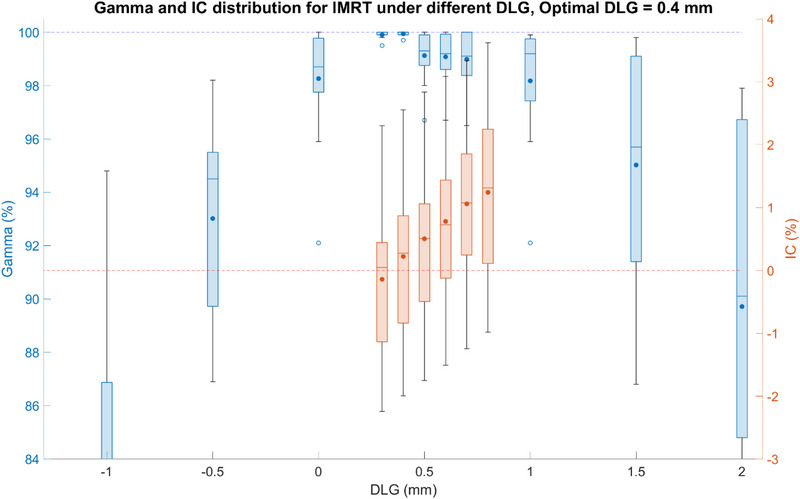
The 3D gamma passing rate between primary TPS and Mobius3D and ion chamber agreement distribution between measurement and Mobius3D under different DLG correction settings for IMRT plans. The blue plots represent the 3D gamma passing rate and the red plots are IC agreement distribution. DLG, dosimetric leaf gap; IC, ion chamber; IMRT, intensity modulated radiation therapy; TPS, treatment planning systems.

IMRT measurement results are shown in Figure 3. The best‐fit DLG correction for IMRT was 0.4 mm, where the mean and standard deviation of ion chamber results are 0.2% ±  1.4% and the mean and standard deviation of 3D gamma is 99.9% ±  0.1% with a range of [99.7%, 100.0%]. When DLG = 0.4 mm, the minimum gamma passing rate is 99.7% and the maximum ion chamber disagreement is 2.6%. The mean and standard deviation of ion chamber measurements to primary TPS is 0.0% ±  1.5% and the median is 0.0%.

Linear regression models of DLG correction as a function of the ion chamber percent difference between measurement and Mobius3D, and gamma passing rate between primary TPS and Mobius3D gave an optimal DLG in mm for VMAT –0.3 (*p* = 0.000) and an optimal DLG in mm for IMRT 0.4 (*p* = 0.003).

## 4 DISCUSSION

Our study results showed that Mobius3D DLG correction should be configured separately for IMRT and VMAT for Ethos to maximize agreement with primary TPS calculated doses and IC measurements. All results were within our institutional guidelines and recommendations of the American Association of Physicists in Medicine Task Group 218,^16^ Task Group 219,^17^ and Medical Physics Practice Guideline 5.b.^8^

For CTgART, the secondary dose calculation is one crucial part of both pre‐treatment and online treatment QA process. To provide a robust check of the adapted plans, good agreement between the secondary check and primary TPS is vital. As can be seen in Figures 2 and 3, if no correction from the default model is applied (DLG correction = 0), the minimum 3D gamma values for VMAT and IMRT are 90.9% and 92.1%. When compared to the DLG correction value applied, the minimum 3D gamma values for VMAT and IMRT are 96.9% and 99.7%. Utilizing the default model can thus potentially result in false positive failures or the need to loosen the warning and tolerance limits resulting in the potential for an increase in false negative situations. Optimizing the DLG correction reduces the chance of both of these scenarios.

The four VMAT ion chamber measurements between 3% and 5% agreement with Mobius3D require future investigation. The treatment sites include accelerated partial breast irradiation (APBI), multiple lesions in abdominal wall, para‐aortic node, and bladder. They are complex plans that have small field components or heavy modulations. The differences between primary TPS calculated doses and measured doses for all 4 plans were all below 1.5%. There are several possible explanations for this discrepancy, which have been studied before in previous literature. There are previous studies^18^, ^19^ that investigated small‐field dosimetry in Mobius3D for TrueBeam (Varian Medical Systems, Inc., Palo Alto, CA). Others^20^, ^21^ reported on the dose uncertainties in small‐field VMAT or off‐axis fields in Mobius3D for Versa and Infinity with Agility MLCs (Elekta, Stockholm, Sweden). It has been shown that Mobius3D accuracy is limited in small fields compared to Eclipse calculation or measurements. Hillman et al. showed Mobius3D can have –21.27% and –8.76% for $0.5\;\times 0.5\;cm^{2}$ and $1\;\times 1\;cm^{2}$ output factor difference compared to Exradin W1 plastic scintillator detector (PSD, Standard Imaging) for high definition multileaf collimator after tuning.^18^, ^19^ They also observed such a difference could be as high as –102.02% for $0.5\;\times 0.5\;cm^{2}$ if Mobius3D is not tuned by the user, that is, the original out‐of‐the‐box model. Small field beam modeling whether for primary dose calculation or for secondary dose calculation remains a topic for further research.^22^ Other contributing factors like plan complexity^23^, ^24^ could also impact the Mobius3D calculation accuracy. Future work will focus on assessing the impact of plan complexity on secondary dose calculation accuracy with a larger sample size.

Although ion chamber measurements are susceptible to setup uncertainties, affected by the choice of point selection, suffer from volume averaging, and limited in describing and assessing the broader 3D dosimetry, they are still a commonly utilized patient‐specific verification method. Measurement‐based PSQA remains a powerful tool for catching inaccuracies of the gantry, collimator, couch, and MLCs.^16^ For example, in this study, ion chamber measurements were able to show the shortcomings of secondary dose calculation. Therefore, combining secondary calculations and measurement‐based PSQA is crucial for process control of safety in treatments.

The choice of 3D gamma passing rate in combination with ion chamber point measurement for tuning the DLG correction parameter in Mobius3D was purposeful so that when 3D gamma results were similar, ion chamber measurement could be used to find the best match. Although it begs to challenge the independence of the secondary calculation software by using the primary dose gamma as one of the input predictors for optimal DLG, the primary TPS uses the golden beam model. Therefore, it is not error‐prone compared to user customization of machine‐specific beam model. Nonetheless, the importance of the ion chamber measurement should be emphasized that it plays an important role in the final optimal DLG determination. This approach provides a more robust final result and has been explored previously.^25^ The recommended DLG correction tuning methodology provided by the vendor guidelines utilizes a linear regression model based on cylindrical ion chamber measurements.^9^ As further verification, we performed this procedure with the same dataset in this study and arrived at the same result. This provides confidence in the community for clinics with fewer resources to perform only ion chamber‐based tuning.

The DLG correction tuning result in this study is specific for our clinic's patient cohort. Clinics should tune their DLG corrections for their specific patient population. Although plan checks cannot completely rely on secondary check software, it remains a powerful tool for identifying errors before PSQA or treatment in the case of CTgART where PSQA cannot be done online.

## 5 CONCLUSION

This study reports single‐institution experiences in tuning the DLG correction parameters for Mobius3D on the Ethos machine for the separate IMRT and VMAT delivery techniques. The configured Mobius3D calculation agreed well with Eclipse calculation and ion chamber measurement results. The results from the study indicate clinics should tune their DLG correction values for each delivery technique separately for their secondary dose calculation algorithms for a more robust CTgART online QA program.

## AUTHOR CONTRIBUTIONS

**Xiaodong Zhao**: Made substantial contributions to the conception and design of the work; the acquisition; analysis; and interpretation of data for the work; drafting the work and revising it critically for important intellectual content; gave final approval of the version to be published; and agrees to be accountable for all aspects of the work in ensuring that questions related to the accuracy or integrity of any part of the work are appropriately investigated and resolved. **Markus Baur**: Made substantial contributions to the conception and design of the work; the acquisition; analysis; and interpretation of data for the work; drafting the work and revising it critically for important intellectual content; gave final approval of the version to be published; and agrees to be accountable for all aspects of the work in ensuring that questions related to the accuracy or integrity of any part of the work are appropriately investigated and resolved. **Phillip D. H. Wall**: Made substantial contributions to the conception and design of the work; the acquisition; analysis; and interpretation of data for the work; drafting the work and revising it critically for important intellectual content; gave final approval of the version to be published; and agrees to be accountable for all aspects of the work in ensuring that questions related to the accuracy or integrity of any part of the work are appropriately investigated and resolved. **Eric Laugeman**: Made substantial contributions to the conception and design of the work; the acquisition; analysis; and interpretation of data for the work; drafting the work and revising it critically for important intellectual content; gave final approval of the version to be published; and agrees to be accountable for all aspects of the work in ensuring that questions related to the accuracy or integrity of any part of the work are appropriately investigated and resolved.

## CONFLICT OF INTEREST STATEMENT

The authors declare no conflicts of interest.

## Data Availability

The data that support the findings of this study are available from the corresponding author upon reasonable request.
